# Differential Regulation of the Immune Response in the Spleen and Liver of Mice Infected with *Leishmania donovani*

**DOI:** 10.1155/2012/639304

**Published:** 2011-07-21

**Authors:** Rashmi Bankoti, Simona Stäger

**Affiliations:** ^1^Department of Molecular and Comparative Pathobiology, The Johns Hopkins University School of Medicine, Baltimore, MD 21205, USA; ^2^Institut National de la Recherche Scientifique, Institut Armand-Frappier, Laval, QC, Canada

## Abstract

Immunity to pathogens requires generation of effective innate and adaptive immune responses. *Leishmania donovani* evades these host defense mechanisms to survive and persist in the host. A better understanding and identification of mechanisms that *L. donovani* employs for its survival is critical for developing novel therapeutic interventions that specifically target the parasite. This paper will highlight some of the mechanisms that the parasite utilizes for its persistence and also discuss how the immune response is regulated.

## 1. Introduction

Visceral leishmaniasis (VL) is caused by the intracellular parasites *Leishmania donovani* and/or *Leishmania infantum/chagasi*. In the mouse model of visceral leishmaniasis, there is a distinct organ-specific pattern of parasite growth during the disease progression. Infection in the liver is characterized by a rapid increase in the parasite burden in the first 4 weeks of infection followed by clearance of the parasite within 6–8 weeks. This self-curing mechanism in the liver is attributed to the development of a Th1 dominated granulamatous response [1] characterized by high IFN*γ* production by CD4 and CD8 T cells. In contrast to liver, infection in the spleen has serious consequences demonstrated by increased parasite burden, disruption of splenic microarchitecture and impaired immune responses resulting in the establishment of parasite persistence [2]. Although the exact mechanism by which the parasite establishes chronic infections in the spleen still remains elusive, it is now becoming evident that the parasite targets and alters the functions of host immune system for evasion. Some of the mechanisms that are altered include suppression of host protective Th1 responses, generation of defective CD8 T cells and inhibition of dendritic cell (DC) functions [2–4]. In addition to modifying DC and T-cell function, the parasite also modulates B-cell function for its survival. Furthermore, by directly interacting with different cellular subsets, the parasite also generates an immunosuppressive environment by inducing IL-10 production and thus favoring its survival in the host. In the first part of this paper we will discuss the above mentioned mechanisms utilized by the parasite to evade host immune response and establish chronic infection in the spleen. The second part of the paper will focus on the *L. donovani* infection in the liver and the regulation of the inflammatory response in this organ.

## 2. Infection in the Spleen

In the experimental model of VL, the spleen is a site of chronic inflammation, characterized by parasite persistence. Chronic infection of the spleen is associated with splenomegaly and changes in the splenic microarchitecture [2, 5, 6], which have consequences on the generation of the immune response to the parasite [5–8]. Typically, following encounter with antigen, dendritic cells (DCs) migrate from the marginal zone (MZ) of the spleen to the T-cell-rich area known as periarteriolar lymphoid sheaths (PALS). In the PALS, the DCs interact with the T cells, and this interaction results in the induction of an antigen-specific T cell response [9]. During chronic *L. donovani* infection, increased production of TNF by macrophages results in the disruption of the splenic MZ [5]. As a consequence of this disruption, DCs [2] and naïve T-cells [5] fail to migrate to the PALS, which may results in diminished priming of T-cells. DCs not only show an impaired migratory capacity, but they also down regulate important costimulatory molecules and increasingly express inhibitory molecules [3]. Hence, it appears that DC functions are defective at later stages of infection. This hypothesis is supported by the fact that adoptive transfer of LPS-activated bone marrow-derived DCs into mice at d21 p.i. results in a significant reduction of the splenic parasite burden [2, 10] and possibly in the induction of protective T cell responses. Splenomegaly is also associated with destruction of the follicular DC network [11] and the gp38^+^ fibroblastic reticular cell network [2]. This disruption is particularly critical since treating or blocking it increases the frequency of protective IFN*γ*-producing CD4 T cells [12]. Conventional CD11c^hi^ splenic DCs not only show impaired migratory capacity during chronic VL, but also increasingly upregulate the inhibitory molecule B7-H1 during chronic infection [3]. B7-H1 is constitutively expressed on subsets of macrophages, thymocytes, and B cells [13], but its expression can also be induced on DCs, epithelial, and endothelial cells [13, 14]. B7-H1 binds to its receptor PD-1 and inhibits T cell proliferation and cytokine production [15]. Furthermore, B7-H1 can also induce programmed cell death of effector T cells by ligation to a yet unknown receptor [16]. During chronic VL, antigen-specific CD8 T cells gradually upregulate PD-1 and display signs of exhaustion characterized by the loss of IL-2, TNF, and finally IFN*γ* production. These functionally exhausted cells eventually die [3]. Exhausted CD8 T cells have also been recently observed in patients with diffuse cutaneous leishmaniasis [17]. The functional exhaustion and deletion of antigen-specific CD8 T cells is in part induced by the upregulation of B7-H1 on splenic DCs. Indeed, in vivo blockade of B7-H1 during chronic *L. donovani* infection results in increased survival of antigen-specific CD8 T cells and also partially restores the functional capacity of exhausted CD8 T cells [3]. The fact that CD8 T-cell functions are not completely restored by B7-H1 blockade suggests the involvement of additional pathways in the suppression of cytokine production by these cells. Other inhibitory molecules, such as LAG3 [18–20] and CTLA4 [21, 22], may also be involved in this suppression. Nevertheless, the partial recovery of CD8 T cell functions is sufficient to reduce the splenic parasite burden, suggesting that B7-H1 blockade and the reactivation of CD8 T-cells may be exploited as a therapeutic intervention.

Other costimulatory and inhibitory molecules were also shown to play a suppressive role during acute experimental VL. Indeed, blocking of either B7-2 [23], OX40, or CTLA-4 [24, 25] results in improved T-cell responses in* L. donovani* infections. Taken together, these results highlight the crucial role of DC during VL and imply that manipulation of the DC response to the parasite may be beneficial for future therapeutic interventions.

Another major factor contributing to progression of disease in VL is IL-10 [10, 26–28]. The role of IL-10 in augmenting disease has been demonstrated by studies that show that IL-10 receptor blockade aids in abrogation of the disease [26]. Additionally, *Il10^−/−^* mice are highly resistant to VL [27]. During chronic infection in mice, IL-10 is mainly expressed by CD25^−^FoxP3^−^CD4^+^ T cells [10], which also produce IFN*γ*. These cells have also been identified in human patients and their presence correlates with disease progression [10]. Although there is evidence linking IL-10 and susceptibility to *L. donovani* infection in humans, inter-individual differences in IL-10 production might contribute to variation in susceptibility to *L. donovani* [29]. Using transgenic mice expressing human IL-10 (*Il10^−/−^*/hIL10BAC), we could investigate the role of cellular-specific IL-10 production on susceptibility to infection. Interestingly, in these transgenic mice IL-10 was properly regulated in the myeloid compartment and resulted in the rescue of *Il10^−/−^* mice from LPS toxicity. However, transgenic IL-10 was weakly expressed in T cells resulting in resistance to *L. donovani* infection, indicating that T-cell-derived IL-10 inhibits the control of parasite burden in the spleen and liver. Thus, T-cell-derived IL-10 plays an essential role in the establishment of chronic infection [30].

IL-10 also plays an important suppressive role during the early stages of infection. We have recently demonstrated that IL-10 contributes to suppression of CD8 T-cell expansion during the early stages of infection (Bankoti et al., submitted). IL-10 blockade also resulted in increased effector functions in CD8 and CD4 T cells. Several cell populations have been identified that upregulate IL-10 production during *L. donovani* infection. These include CD4 T cells [10], NK cells [31], macrophage [26], and DCs [32], and potentially one of these cell populations could be responsible for suppression of CD8 T-cell expansion and CD8 and CD4 T-cell function. However, data from our laboratory suggest that there is probably no major source of IL-10 responsible for the suppression of T-cell function, but several IL-10 sources contribute to it (Bankoti and Stäger, unpublished). 

Several studies indicate that B cells have an adverse effect on disease outcome in various models of leishmaniasis [33–36]. In experimental VL, B cells also contribute to disease exacerbation. Indeed, in the absence of B cells, mice are resistant to *L. donovani* infection [35]. The mechanism by which B cells contribute to parasite persistence has not yet been completely defined. One of the possible mechanisms by which B cells exacerbate disease is by secretion of antibodies. The role of antibodies in progression of disease was demonstrated by using J_H_ mice that produce no antibodies. Infection of *J_H_* mice with *L. major* resulted in smaller lesions and limitation of the parasite burden. However, reconstituting J_H_ mice with anti-*L*.* major* sera at d21 postinfection resulted in exacerbation of the disease [37, 38]. This increased susceptibility to infection could be explained by the fact that host IgG on the amastigote surface ligates the macrophage Fc*γ*Rs and induces IL-10 production by macrophages [37]. One of the consequences of this increased IL-10 production is the inactivation of macrophages which further contributes to parasite persistence. In addition to IgG, increased IgM production and polyclonal B-cell activation has also been recently demonstrated as a cause of disease exacerbation in *L. infantum* infected mice during the early stages of infection [34]. 

Our recent findings suggest that, in addition to inducing IL-10 production by macrophages via antibody secretion [37], B cells directly interact with* L. donovani* and this interaction results in the suppression of protective T-cell responses in an antibody-independent manner. Following i.v. injection, amastigotes move to the spleen where they initially come in contact with cells of the marginal zone (MZ). The amastigotes are primarily internalized by marginal zone macrophages (MZM) and metallophilic macrophages (MM) present in the MZ of the spleen [4, 9]. The marginal zone also consists of the marginal zone B cells (MZBs) [39] that are located strategically to encounter the parasite early on during infection. Marginal zone B cells are noncirculating B cells and initiate T-independent responses in response to blood borne pathogens [40, 41]. MZBs also have the capacity to rapidly differentiate into short lived IgM producing antibody forming plasma cells [42]. We recently observed that *L. donovani* closely interacts with B cells in vivo, inducing activation and cluster formation (Bankoti et al., submitted). We also observed an association between amastigotes and cells that displayed an MZB phenotype in vivo, 24hr after infection, suggesting that MZBs capture parasites during the very early stages of disease. Although, the exact mechanism of parasite capture remains unclear, it is possible that MZB cells recognize amastigotes through surface IgM and/or through the complement receptor 2 (CD21). This interaction of the parasite and MZBs resulted in the upregulation of the costimulatory molecules CD80 and CD86 and the production of IL-10 by two B-cell subpopulations: marginal zone B-cell-like cells and regulatory B-cells-like cells (Bankoti et al., submitted). Moreover, in agreement with previous report [34], B cells upon interacting with *L. donovani* increased surface IgM expression and secrete IgM (Bankoti et al. submitted). In addition to inducing IL-10 production by MZB cells, cross-linking of CD21 also induces the migration of MZBs to the white pulp [41, 43, 44]. Hence, it is possible that the MZB cells capture the parasite and migrate to the white pulp where they can then transfer the antigen to the follicular DCs [45–47]. Follicular DCs can then retain the antigen for a long duration of time and initiate CD4 and CD8 T-cell responses. Follicular DCs can also interact with the follicular B cells and result in polyclonal B-cell activation [48], which is typically observed during human VL [49–52]. Activation of MZBs by *L. donovani* is detrimental to the course of infection, since depletion of MZB results in significantly lower splenic parasite burdens and in stronger CD8 and CD4 T-cell responses. This suggests that marginal zone B cells suppress protective T-cell responses during early stages of *L. donovani *infection and contribute to the establishment of chronic disease. Although MZBs secrete IL-10, this suppressive effect is only partially mediated by MZB-derived IL-10. Thus, it is possible that MZB inhibit APC functions and/or induce IL-10 production by other cell populations, such as macrophages or T cells. Further investigations are needed to test this hypothesis.

IL-10 production by B cells was also shown to induce Th2 responses in a model of cutaneous leishmaniasis [53]. B cells also produce IL-10 upon encounter with *L. infantum* [34]; however, this B-cell-derived IL-10 does not seem to affect the parasite burden at least at d28 p.i. [34].

## 3. Infection in Liver

The liver is another main target organ in the experimental model of VL. In contrast to the spleen that stays chronically infected, infection in the liver is self-resolving within 6–8 weeks. Resolution of disease in the liver is associated to the development of granuloma formation which is one of the key features of hepatic resistance [54]. The resolution of the infection in the liver is attributed to the development of a Th1-dominated granulomatous response, characterized by high IFN*γ* production by CD4 T cells. The Th1 response in turn is initiated by IL-12 secreted from the DCs [9, 55]. IL-12 is an essential cytokine in the development of protective immunity to *L. donovani,* since blocking of IL-12 reduced both the IFN*γ* production and granuloma formation in the liver of infected mice [56]. Furthermore, supplementing exogenous IL-12 to *L. donovani*-infected mice early during infection decreased the liver parasite burden [57]. CD8 T cells also have an essential role in the clearance of the parasite in the liver. Indeed, depletion of CD8 T cells inhibits the development of the granulomatous response and results in disease exacerbation [1]. Moreover, CD8 T cells have been shown to be mediators of resistance to rechallenge [58]. In addition to the adaptive T-cell response, TNF production and expression of inducible nitric oxide synthase (iNOS or NOS2) by macrophages aid in clearing the infection. Hepatic resistance in VL is also attributed to the generation of reactive nitrogen and oxygen intermediates, both of which have been shown to play a role in containing parasite growth during the early stages of infection [59]. These might in turn be related to the T-cell-dependent recruitment of monocytes at these early time points [60]. At the later stage of infection, iNOS gene regulation appears to play an important role and generation of NO indicates T-cell-dependent macrophage activation [60]. 

Initiation of the immune response requires the recognition of the parasite by the immune system. The presence of certain molecules on the pathogens, known as pathogen-associated molecular patterns (PAMPs) allows the immune system to recognize the pathogen and elicit an immune reaction. These PAMPs are recognized by Toll-like receptors (TLRs) present on the antigen presenting cells. Recognition of a pathogen by TLRs initiates a signaling cascade that ultimately results in the induction of cytokines. The interferon regulatory factors (IRFs) are important parts of this signaling chain. One of the IRFs, IRF5, is of particular interest and has previously been demonstrated to induce proinflammatory cytokines such as IL-12, TNF, and IL-6 in response to viruses [61, 62]. This was further supported by the fact that in *Irf5^−/−^* mice there is attenuation of type I IFNs, TNF and IL-6 production in response to viral infection [63–65]. We have recently demonstrated that *L. donovani* elicits a defective Th1 responses in the liver of *Irf5^−/−^* mice and results in fewer liver granuloma and exacerbation of the hepatic infection [66]. Additionally, the granulomas in *Irf5^−/−^* mice were also considerably smaller in size suggesting a defective recruitment of inflammatory cells into the liver of *Irf5^−/−^* mice. These results emphasize the importance of IRF5 as an essential transcription factor to initiate the inflammatory response following *L. donovani* infection. Recently, IRF5 was shown to promote inflammatory macrophage polarization [67]. M1 macrophages, also known as the classically activated macrophages, are induced by IFN*γ* to produce proinflammatory cytokines, for example, TNF, IL-6, and IL-23. In addition to producing pro-inflammatory cytokines, M1 macrophages also exhibit enhanced microbicidal activity. M2 macrophages on the other hand are known as alternatively activated macrophages, which produce anti-inflammatory cytokine and enhance tissue repair [68, 69]. Interestingly, IRF5 influences the polarization of macrophages. M1 macrophages have high IRF5 expression and therefore activate genes for IL-12p40, IL-12p35, and IL-23p19 and repress IL-10 gene [67]. Classically activated macrophages upon triggering of interferon and TLR pathway also increase expression of iNOS that results in production of NO [70–72]. Hence, it is not surprising that iNOS protein is not produced in the granulomas of *Irf5^−/−^* mice and that *Irf5^−/−^* mice are more susceptible to VL. The exact pathway triggered by *L. donovani* that results in the induction of IRF5 and the mechanism by which IRF5 induces the inflammatory response during *L. donovani* infection are still undefined. IRF5 is downstream of TLR7 and 9, but can also be directly induced by type I IFN [73]. Thus, one could speculate that *L. donovani* may trigger TLR7 and/or TLR9 and induce IRF5 expression. It would be interesting to examine whether IFR5 is induced by *L. donovani* in *Tlr9^−/−^Tlr7^−/−^* double-knockout mice and whether TLR7 and TLR9 have any overlapping functions. IRF5 upregulation appears to be required by different cells at different time of infection. For instance, the absence of IRF5 does not seem to affect the early development of Th1 responses. In contrast, this transcription factor is essential for the maintenance of Th1 responses and is increasingly expressed in T cells during chronic infection. Conversely, IRF5 deficiency severely impaired the infiltration of inflammatory cells in the liver already at earlier stages of infection. How this timely expression of IRF5 regulates the immune response and the role of IRF5 in T cells are still some of the questions that remain unanswered and need further investigations. Several studies have demonstrated the importance of other IRFs during *L. donovani* infection. *L. donovani* has been shown to induce IRF-binding activity in macrophages *in vivo* [74]. Furthermore, IRF7 was shown to have a role in regulating the killing of parasites by splenic marginal zone macrophage [75]. Additionally, *L. donovani* amastigotes disrupt the interaction between STAT1*α* and importin-*α*5 (a nuclear transport adaptor protein) resulting in the inhibition of IFN*γ*-induced expression of IRF1 in macrophages [76]. Although these studies reveal the importance of IRFs as an important regulator of signaling events and modulating the outcome of disease, further investigations are required to completely understand the exact mechanisms of IRF-induced pathways to use it for therapeutic purposes in *L. donovani* infections.

## 4. Conclusion

*Leishmania donovani* utilizes several mechanisms to evade the host immune system depending on the cell that it interacts with during the course of infection. Although these events might not be chronologically linked, clearly the parasite encounters different cells of the immune system and efficiently evades its clearance. Upon intravenous infection with *L. donovani* in mice, the cells that first come in contact with the parasite in the spleen are DCs, macrophages, and the MZ B cells. The parasite evades these immune cells by either interfering with proper DC function or by inducing IL-10 production by macrophages, B and T cells, resulting in an immunosuppressive environment. The effect of these altered functions early during the disease is reflected by generation of defective CD8 T and CD4 T-cell responses. Clearly there is a dearth of knowledge regarding the complex mechanism that the parasite utilizes to evade the immune system, and a better understanding of the complex interaction of various cell types with the parasite is needed to aid in development of novel therapeutics interventions.

## References

[1] Stern JJ, Oca MJ, Rubin BY, Anderson SL, Murray HW (1988). Role of L3T4+ and Lyt-2+ cells in experimental visceral leishmaniasis. *Journal of Immunology*.

[2] Ato M, Stäger S, Engwerda CR, Kaye PM (2002). Defective CCR7 expression on dendritic cells contributes to the development of visceral leishmaniasis. *Nature Immunology*.

[3] Joshi T, Rodriguez S, Perovic V, Cockburn IA, Stäge S (2009). B7-H1 blockade increases survival of dysfunctional CD8(+) T cells and confers protection against Leishmania donovani infections. *PLoS Pathogens*.

[4] Ato M, Maroof A, Zubairi S, Nakano H, Kakiuchi T, Kaye PM (2006). Loss of dendritic cell migration and impaired resistance to Leishmania donovani infection in mice deficient in CCL19 and CCL21. *Journal of Immunology*.

[5] Engwerda CR, Ato M, Cotterell SEJ (2002). A role for tumor necrosis factor-*α* in remodeling the splenic marginal zone during Leishmania donovani infection. *American Journal of Pathology*.

[6] Stanley AC, Engwerda CR (2007). Balancing immunity and pathology in visceral leishmaniasis. *Immunology and Cell Biology*.

[7] Kaye PM, Svensson M, Ato M (2004). The immunopathology of experimental visceral leishmaniasis. *Immunological Reviews*.

[8] Engwerda CR, Kaye PM (2000). Organ-specific immune responses associated with infectious disease. *Immunology Today*.

[9] Gorak PM, Engwerda CR, Kaye PM (1998). Dendritic cells, but not macrophages, produce IL-12 immediately following Leishmania donovani infection. *European Journal of Immunology*.

[10] Stager S, Maroof A, Zubairi S, Sanos SL, Kopf M, Kaye PM (2006). Distinct roles for IL-6 and IL-12p40 in mediating protection against Leishmania donovani and the expansion of IL-CD4^+^CD25^+^ T cells. *European Journal of Immunology*.

[11] Smelt SC, Engwerda CR, McCrossen M, Kaye PM (1997). Destruction of follicular dendritic cells during chronic visceral leishmaniasis. *Journal of Immunology*.

[12] Dalton JE, Maroof A, Owens BMJ (2010). Inhibition of receptor tyrosine kinases restores immunocompetence and improves immune-dependent chemotherapy against experimental leishmaniasis in mice. *Journal of Clinical Investigation*.

[13] Greenwald RJ, Freeman GJ, Sharpe AH (2005). The B7 family revisited. *Annual Review of Immunology*.

[14] Chen L (2004). Co-inhibitory molecules of the B7-CD28 family in the control of T-cell immunity. *Nature Reviews Immunology*.

[15] Freeman GJ, Long AJ, Iwai Y (2000). Engagement of the PD-1 immunoinhibitory receptor by a novel B7 family member leads to negative regulation of lymphocyte activation. *Journal of Experimental Medicine*.

[16] Dong H, Strome SE, Salomao DR (2002). Tumor-associated B7-H1 promotes T-cell apoptosis: a potential mechanism of immune evasion. *Nature Medicine*.

[17] Hernández-Ruiz J, Salaiza-Suazo N, Carrada G (2010). CD8 cells of patients with diffuse cutaneous leishmaniasis display functional exhaustion: the latter is reversed, in vitro, by TLR2 agonists. *PLoS Neglected Tropical Diseases*.

[18] Grosso JF, Goldberg MV, Getnet D (2009). Functionally distinct LAG-3 and PD-1 subsets on activated and chronically stimulated CD8 T cells. *Journal of Immunology*.

[19] Workman CJ, Cauley LS, Kim IJ, Blackman MA, Woodland DL, Vignali DAA (2004). Lymphocyte activation gene-3 (CD223) regulates the size of the expanding T cell population following antigen activation in vivo. *Journal of Immunology*.

[20] Workman CJ, Vignali DAA (2003). The CD4-related molecule, LAG-3 (CD223), regulates the expansion of activated T cells. *European Journal of Immunology*.

[21] Favali C, Costa D, Afonso L (2005). Role of costimulatory molecules in immune response of patients with cutaneous leishmaniasis. *Microbes and Infection*.

[22] Khan S, Burt DJ, Ralph C, Thistlethwaite FC, Hawkins RE, Elkord E (2010). Tremelimumab (anti-CTLA4) mediates immune responses mainly by direct activation of T effector cells rather than by affecting T regulatory cells. *Clinical Immunology*.

[23] Murphy ML, Engwerda CR, Gorak PMA, Kaye PM (1997). B7-2 blockade enhances T cell responses to Leishmania donovani. *Journal of Immunology*.

[24] Zubairi S, Sanos SL, Hill S, Kaye PM (2004). Immunotherapy with OX40L-Fc or anti-CTLA-4 enhances local tissue responses and killing of Leishmania donovani. *European Journal of Immunology*.

[25] Murphy ML, Cotterell SEJ, Gorak PMA, Engwerda CR, Kaye PM (1998). Blockade of CTLA-4 enhances host resistance to the intracellular pathogen, Leishmania donovani. *Journal of Immunology*.

[26] Murray HW, Lu CM, Mauze S (2002). Interleukin-10 (IL-10) in experimental visceral leishmaniasis and IL-10 receptor blockade as immunotherapy. *Infection and Immunity*.

[27] Murphy ML, Wille U, Villegas EN, Hunter CA, Farrell JP (2001). IL-10 mediates susceptibility to Leishmania donovani infection. *European Journal of Immunology*.

[28] Belkaid Y, Piccirillo CA, Mendez S, Shevach EM, Sacks DL (2002). CD4^+^CD25^+^ regulatory T cells control Leishmania major persistence and immunity. *Nature*.

[29] Nylén S, Sacks D (2007). Interleukin-10 and the pathogenesis of human visceral leishmaniasis. *Trends in Immunology*.

[30] Ranatunga D, Hedrich CM, Fengying W (2009). A human IL10 BAC transgene reveals tissue-specific control of IL-10 expression and alters disease outcome. *Proceedings of the National Academy of Sciences of the United States of America*.

[31] Maroof A, Beattie L, Zubairi S, Svensson M, Stager S, Kaye PM (2008). Posttranscriptional regulation of II10 gene expression allows natural killer cells to express immunoregulatory function. *Immunity*.

[32] Svensson M, Maroof A, Ato M, Kaye PM (2004). Stromal cells direct local differentiation of regulatory dendritic cells. *Immunity*.

[33] Sacks DL, Scott PA, Asofsky R, Sher FA (1984). Cutaneous leishmaniasis in anti-IgM-treated mice: enhanced resistance due to functional depletion of a B cell-dependent T cell involved in the suppressor pathway. *Journal of Immunology*.

[34] Deak E, Jayakumar A, Cho KW (2010). Murine visceral leishmaniasis: IgM and polyclonal B-cell activation lead to disease exacerbation. *European Journal of Immunology*.

[35] Smelt SC, Cotterell SEJ, Engwerda CR, Kaye PM (2000). B cell-deficient mice are highly resistant to Leishmania donovani infection, but develop neutrophil-mediated tissue pathology. *Journal of Immunology*.

[36] Ronet C, Hauyon-La Torre Y, Revaz-Breton M (2010). Regulatory B cells shape the development of Th2 immune responses in BALB/c mice infected with Leishmania major through IL-10 production. *Journal of Immunology*.

[37] Miles SA, Conrad SM, Alves RG, Jeronimo SMB, Mosser DM (2005). A role for IgG immune complexes during infection with the intracellular pathogen Leishmania. *Journal of Experimental Medicine*.

[38] Kane MM, Mosser DM (2001). The role of IL-10 in promoting disease progression in Leishmaniasis. *Journal of Immunology*.

[39] Martin F, Kearney JF (2002). Marginal-zone B cells. *Nature Reviews Immunology*.

[40] Lopes-Carvalho T, Kearney JF (2004). Development and selection of marginal zone B cells. *Immunological Reviews*.

[41] Balazs M, Martin F, Zhou T, Kearney JF (2002). Blood dendritic cells interact with splenic marginal zone B cells to initiate T-independent immune responses. *Immunity*.

[42] Oliver AM, Martin F, Kearney JF (1999). IgM(high)CD21(high) lymphocytes enriched in the splenic marginal zone generate effector cells more rapidly than the bulk of follicular B cells. *Journal of Immunology*.

[43] Cinamon G, Zachariah MA, Lam OM, Foss FW, Cyster JG (2008). Follicular shuttling of marginal zone B cells facilitates antigen transport. *Nature Immunology*.

[44] Whipple EC, Shanahan RS, Ditto AH, Taylor RP, Lindorfer MA (2004). Analyses of the in vivo trafficking of stoichiometric doses of an anti-complement receptor 1/2 monoclonal antibody infused intravenously in mice. *Journal of Immunology*.

[45] Suzuki K, Grigorova I, Phan TG, Kelly LM, Cyster JG (2009). Visualizing B cell capture of cognate antigen from follicular dendritic cells. *Journal of Experimental Medicine*.

[46] Ferguson AR, Youd ME, Corley RB (2004). Marginal zone B cells transport and deposit IgM-containing immune complexes onto follicular dendritic cells. *International Immunology*.

[47] Youd ME, Ferguson AR, Corley RB (2002). Synergistic roles of IgM and complement in antigen trapping and follicular localization. *European Journal of Immunology*.

[48] Rajewsky K (1996). Clonal selection and learning in the antibody system. *Nature*.

[49] Ghose AC, Haldar JP, Pal SC (1980). Serological investigations on Indian kala-azar. *Clinical & Experimental Immunology*.

[50] Pontes de Carvalho LC, Badaro R, Carvalho EM (1986). Nature and incidence of erythrocyte-bound IgG and some aspects of the physiopathogenesis of anaemia in American visceral leishmaniasis. *Clinical & Experimental Immunology*.

[51] Louzir H, Belal-Kacemi L, Sassi A, Laouini D, Ismail RB, Dellagi K (1994). Natural autoantibodies, IgG antibodies to tetanus toxoid and CD5+ B cells in patients with Mediterranean visceral leishmaniasis. The Leishmania study group. *Clinical & Experimental Immunology*.

[52] Galvao-Castro B, Sa Ferreira JA, Marzochi KF (1984). Polyclonal B cell activation, circulating immune complexes and autoimmunity in human American visceral leishmaniasis. *Clinical & Experimental Immunology*.

[53] Ronet C, Voigt H, Himmelrich H (2008). Leishmania major-specific B cells are necessary for Th2 cell development and susceptibility to L. major LV39 in BALB/c mice. *Journal of Immunology*.

[54] Murray HW (2001). Tissue granuloma structure-function in experimental visceral leishmaniasis. *International Journal of Experimental Pathology*.

[55] Scharton-Kersten T, Afonso LCC, Wysocka M, Trinchieri G, Scott P (1995). IL-12 is required for natural killer cell activation and subsequent T helper 1 cell development in experimental Leishmaniasis. *Journal of Immunology*.

[56] Murray HW (1997). Endogenous interleukin-12 regulates acquired resistance in experimental visceral leishmaniasis. *Journal of Infectious Diseases*.

[57] Murray HW, Hariprashad J (1995). Interleukin 12 is effective treatment for an established systemic intracellular infection: experimental visceral leishmaniasis. *Journal of Experimental Medicine*.

[58] Murray HW, Squires KE, Miralles CD (1992). Acquired resistance and granuloma formation in experimental visceral leishmaniasis. Differential T cell and lymphokine roles in initial versus established immunity. *Journal of Immunology*.

[59] Murray HW, Nathan F (1999). Macrophage microbicidal mechanisms in vivo: reactive nitrogen versus oxygen intermediates in the killing of intracellular visceral Leishmania donovani. *Journal of Experimental Medicine*.

[60] Cervia JS, Rosen H, Murray HW (1993). Effector role of blood monocytes in experimental visceral leishmaniasis. *Infection and Immunity*.

[61] Barnes BJ, Richards J, Mancl M, Hanash S, Beretta L, Pitha PM (2004). Global and distinct targets of IRF-5 and IRF-7 during innate response to viral infection. *Journal of Biological Chemistry*.

[62] Barnes BJ, Moore PA, Pitha PM (2001). Virus-specific activation of a novel interferon regulatory factor, IRF-5, results in the induction of distinct interferon *α* genes. *Journal of Biological Chemistry*.

[63] Paun A, Reinert JT, Jiang Z (2008). Functional characterization of murine interferon regulatory factor 5 (IRF-5) and its role in the innate antiviral response. *Journal of Biological Chemistry*.

[64] Yanai H, Chen HM, Inuzuka T (2007). Role of IFN regulatory factor 5 transcription factor in antiviral immunity and tumor suppression. *Proceedings of the National Academy of Sciences of the United States of America*.

[65] Takaoka A, Yanai H, Kondo S (2005). Integral role of IRF-5 in the gene induction programme activated by Toll-like receptors. *Nature*.

[66] Paun A, Bankoti R, Joshi T, Pitha PM, Stäger S (2011). Critical role of IRF-5 in the development of T helper 1 responses to Leishmania donovani infection. *PLoS Pathogens*.

[67] Krausgruber T, Blazek K, Smallie T (2011). IRF5 promotes inflammatory macrophage polarization and T_H_1-T_H_17 responses. *Nature Immunology*.

[68] Mosser DM, Edwards JP (2008). Exploring the full spectrum of macrophage activation. *Nature Reviews Immunology*.

[69] Dale DC, Boxer L, Conrad Liles W (2008). The phagocytes: neutrophils and monocytes. *Blood*.

[70] Thoma-Uszynski S, Stenger S, Takeuchi O (2001). Induction of direct antimicrobial activity through mammalian toll-like receptors. *Science*.

[71] Bogdan C (2001). Nitric oxide and the immune response. *Nature Immunology*.

[72] Brightbill HD, Libraty DH, Krutzik SR (1999). Host defense mechanisms triggered by microbial lipoproteins through toll-like receptors. *Science*.

[73] Schoenemeyer A, Barnes BJ, Mancl ME (2005). The interferon regulatory factor, IRF5, is a central mediator of toll-like receptor 7 signaling. *Journal of Biological Chemistry*.

[74] Balaraman S, Tewary P, Singh VK, Madhubala R (2004). Leishmania donovani induces interferon regulatory factor in murine macrophages: a host defense response. *Biochemical and Biophysical Research Communications*.

[75] Phillips R, Svensson M, Aziz N (2010). Innate killing of Leishmania donovani by macrophages of the splenic marginal zone requires IRF-7. *PLoS Pathogens*.

[76] Matte C, Descoteaux A (2010). Leishmania donovani amastigotes impair gamma interferon-induced STAT1*α* nuclear translocation by blocking the interaction between STAT1*α* and importin-*α*5. *Infection and Immunity*.

